# Volitional spatial attention is lateralized in crows

**DOI:** 10.1098/rspb.2024.2540

**Published:** 2025-01-29

**Authors:** Linus Hahner, Andreas Nieder

**Affiliations:** ^1^ Animal Physiology Unit, Institute of Neurobiology, University of Tübingen, Auf der Morgenstelle 28, Tübingen 72076, Germany

**Keywords:** volitional, spatial, attention, lateralized, crows

## Abstract

Like humans and many other animal species, birds exhibit left–right asymmetries in certain behaviours due to differences in hemispheric brain functions. While the lateralization of sensory and motor functions is well established in birds, the potential lateralization of high-level executive control functions, such as volitional attention, remains unknown. Here, we demonstrate that carrion crows exhibit more pronounced volitional (endogenous) attention for stimuli monocularly viewed with the left eye and thus in the left visual hemifield. We trained four crows on Posner-like spatial cueing tasks using informative cues to evaluate their volitional top-down attention. The crows detected cued targets using either the left or right eye. As a measure of volitional attention, we calculated reaction time differences for detecting targets that were correctly (validly) and incorrectly (invalidly) cued, separately for the left and right visual hemifields. We found that cued targets were detected more quickly and efficiently in the left visual field compared with the right visual field. Because the left-eye system of the crow’s brain processes information primarily from the left visual hemifield, these findings suggest that crows, like humans, exhibit superior executive control of attention in the left-eye/right hemisphere system of their brains.

## 1. Introduction

Brain lateralization captures the finding that specific tasks and abilities are more efficiently managed by either the left- or right-eye system of the brain. Lateralization phenomena are common in diverse species, from insects to humans [1]. In humans, for instance, the left hemisphere is typically associated with language skills, whereas the right hemisphere is generally linked to spatial attentional abilities [2]. It is thought that the division of functions between the eye systems can enhance overall cognitive capacity to handle more information simultaneously [3].

Bird species have been among the first animals in which prominent brain lateralization had been shown in vision, song production and sleep [1,3]. In vision, information primarily reaches the contralateral side of the brain; thus, differences in lateralization can be identified by comparing performance between the left and right eye in birds with laterally placed eyes. For instance, when chicks and pigeons were free to orient and peck at grains spread evenly in front of them, they displayed a notable leftward bias [4,5]. Because such spontaneous foraging tasks involve complex behaviours that integrate sensorimotor components —such as detecting a grain, orienting towards it and pecking for it—they do not allow one to discriminate the precise behavioural mechanisms responsible for side biases.

To distinguish between different types of attention in visuospatial processing, several bird species [6–9] have recently been tested using variants of Posner’s spatial cueing task [10]. In such tasks, the more rapid and precise detection of a validly cued target compared with an invalidly cued target provides evidence for spatial attention. The type of cue preceding target presentation allows differentiation between two fundamental types of attention: stimulus-driven, exogenous (reflexive) attention and top-down, endogenous (volitional) attention [11]. A spatial cue that does not predict the target location examines the effects of automatic exogenous attention. In contrast, an informative cue that predicts the target location allows a subject to cognitively and covertly control their focus of attention, making it suitable for testing endogenous attention effects [12].

Recently, we demonstrated that carrion crows meet the standard criteria for endogenous selective attention [13], an indicator of subjective, conscious experience [14–17]. When presented with predictive spatial cues in a Posner-like task design, they covertly and continuously attend to the cued side at the expense of other locations, resulting in faster reaction times (RTs) and improved performance for valid cues [13,18]. In the current study, the analyses of spatial cueing effects in four carrion crows were extended by comparing their responses to left versus right visual targets. We tested crows using two related protocols: a peripheral cueing task and a central cueing task. In the peripheral cueing task, the predictive cues and potential target positions coincided. In contrast, the central task presented predictive cues spatially separated from the potential target positions, requiring the crows to volitionally orient their attention away from the cue and towards the target. This latter design allowed us to exclude confounding factors such as exogenous attention or visual masking related to cue onset. Ultimately, this approach aimed to investigate the potential lateralization of volitional attention in crows.

## 2. Methods

### (a) Animals

Behavioural data were recorded in four carrion crows (*Corvus corone*), three males (Crows 1−3, 2.5−6 years) and one female (Crow 4, 1.5 years) from the university’s facilities. All the crows tested were bred naturally by their biological parents in their self-constructed nests, hatched in those original nests, and were initially reared by their parents in large outdoor aviaries at the university’s facilities. The crows were kept in social groups in spacious aviaries. During training, crows were kept on a controlled feeding protocol, and food was used as a reward. The crows always had *ad libitum* access to water. All procedures were carried out according to the guidelines for animal experimentation of, and approved by, the responsible authority under national legislation, the Regierungspräsidium Tübingen, Germany.

### (b) Experimental setup

**Figure 1.**
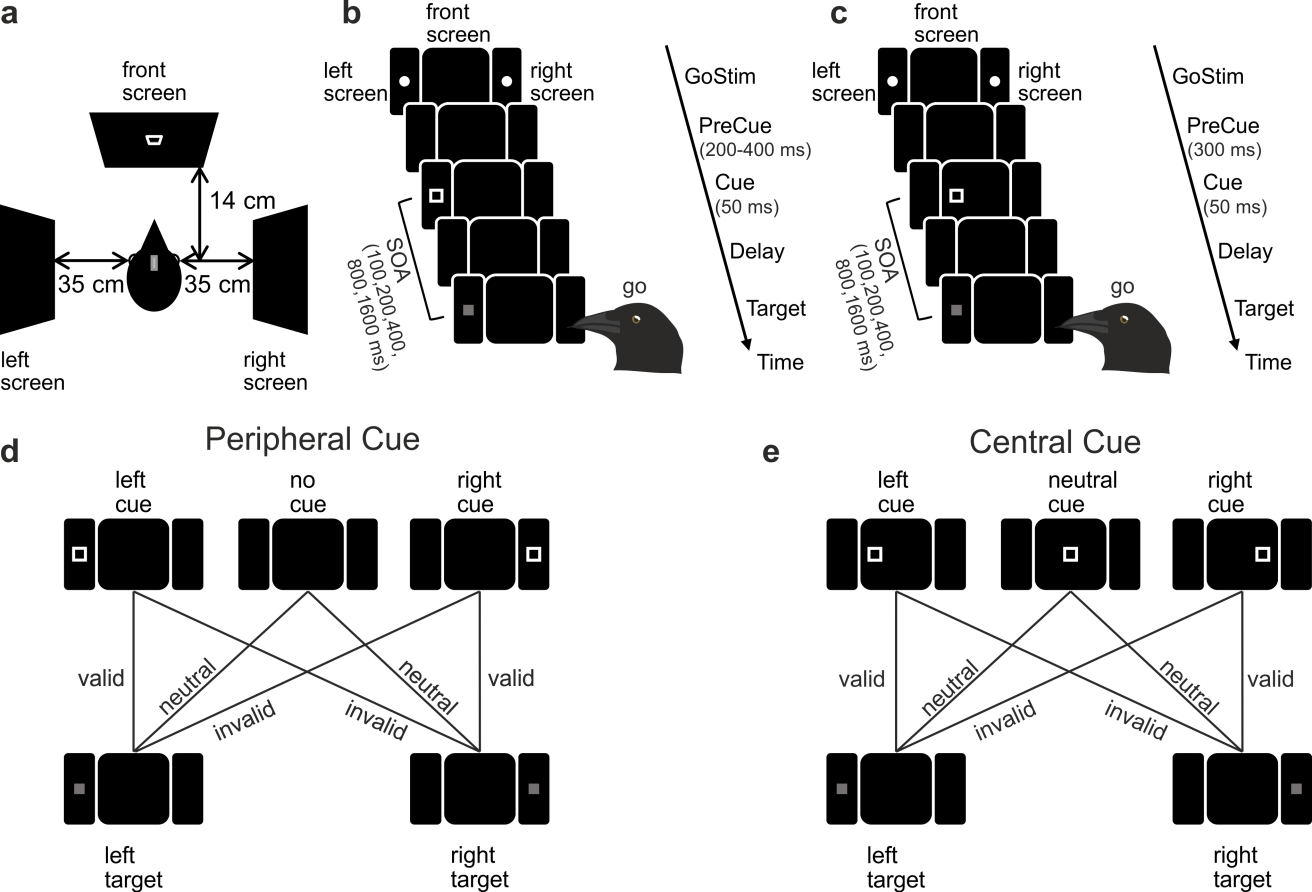
Endogenous cueing task. (a) Experimental set-up seen from above. Crows responded to stimuli presented on a frontal and two lateral screens. (b) Peripheral cueing task. Cue and target stimuli appeared on the left and right-side screen, separated by a variable delay. Crows had to respond to the target within 550 ms. (c) Central cueing task. Identical set-up as in (b), except the cue appeared on the front screen. A cue displaced by 6.5 cm to the left relative to the centre of the front screen signified the occurrence of the target on the left-side screen, whereas a cue displaced to the right predicted the appearance of the target on the right-side screen. (d) Stimulus combinations in the peripheral task. Cue and target side were balanced across conditions. Valid cues appeared on the side of the upcoming target, invalid cues on the opposite side. Spatial cues were 89% valid. In 10% of all trials, no cue was shown. (e) Stimulus combinations in the central task. Valid cues were shifted to the side of the upcoming target, invalid cues to the opposite side. Spatial cues were 87.5% valid. In 20% of all trials, a neutral cue was shown in the middle of the screen.

Training and data acquisition took place in a closed conditioning chamber (102 × 100 × 76 cm) with a side door. The crows sat on a wooden perch 14 cm in front of a touch screen (3M. Microtouch, 15″, 60 Hz refresh rate). On the side walls, two additional screens (Joy-it RB-LCD10-2, 10.1″, 60 Hz refresh rate) were mounted at a 35 cm viewing distance each (figure 1*a*). Below the front screen, the crows could retrieve food rewards from a custom-made automatic feeder. The task flow was controlled online using the CORTEX program (National Institute of Mental Health). Visual stimuli were presented on the front and side screens, and auditory feedback was given by a speaker mounted behind the front screen. The crow’s head position was tracked using a custom-written tracking program in MATLAB. This program used the live feed of two infrared-vision cameras (Body: FLIR CM3-U3-13y3M, Lens: Fujinon DF6HA-1B, IR-Emitter: Kingbright BL0106-15-28, 940 nm) in the chamber. One camera was mounted on the side wall left to the front screen and tracked the vertical plane, the other was mounted on the top wall and tracked the horizontal plane. The crows had to fixate head position throughout the task by keeping a small reflector attached to the top of their head inside of a predetermined area centred between the two side screens. The allowed area for the reflector measured 2.7 × 2.7 × 2.5 cm, and the head angle had to remain within ± 20° of a straight-forward orientation in the horizontal plane. With these constraints, stimuli presented on either side screen could only be perceived monocularly, whereas stimuli presented in front were within the binocular visual-field range of the carrion crows [19]. To indicate detection of the target stimulus, the crows had to break fixation by moving their head outside of the tracking area, whereupon the feeder briefly lit up and delivered a reward.

### (c) Behavioural task

This article constitutes a detailed analysis of behavioural data that were collected from our crows in two previous Posner-like endogenous spatial cueing studies [13,18]. Crows 1−4 participated in a cueing task with peripheral predictive cues [13]. Crows 3 and 4 additionally participated in a cueing task with central predictive cues [18].

### (d) Task 1: peripheral predictive cueing

The general task layout is shown in figure 1*b*. The crows had to detect a visual target on the side screens after being cued about its probable location (left or right) in each trial. The beginning of a trial was indicated by two white circles of 4 mm diameter (0.7° visual angle), the go-stimulus, presented at the two possible target locations. To initiate a trial, the crows had to fixate their head position in the tracking area within 30 s of go-stimulus onset. To complete a trial successfully, they had to maintain fixation up to the response phase. Upon fixation, the go-stimulus disappeared, and all screens remained dark for a precue phase of 200−400 ms. Next, a spatial cue was shown in 90% of all trials. The cue stimulus was a white square outline of 1 mm width covering a 2.95° visual angle. It was presented either on the left- or the right-side screen at the potential target location. In 89% of cued trials, a valid cue was presented on the side of the impending target, predicting its location. In 11% of cued trials, an invalid cue was presented on the side opposite to the impending target. Cue and target sides were balanced across conditions. In 10% of all trials, no cue was shown. The cue phase lasted for 50 ms, followed by a variable delay period. During the delay period, all screens remained dark. Five delay lengths of 50−1550 ms were presented fully balanced. Together with the cue period, this defined five different stimulus-onset asynchronies (SOAs) of 100, 200, 400, 800 and 1600 ms. After the delay, the target stimulus, a grey-filled square of 0.7 mm width (1.15° visual angle), was presented on either side screen. The target had one of three different intensities: 10.2, 3.1 or 0.43 cd m^−^² from highest to lowest. Target side and intensity were fully balanced across conditions. The crows had to report target onset by leaving fixation within 550 ms, usually via a ‘nodding’ movement. Correct responses were rewarded with food and a reward sound, and the RT was measured. If the crows did not respond within 550 ms, the trial was counted as an error and all screens briefly lit up yellow, followed by a 2000 ms timeout before a new trial began. If the crows broke fixation at any time before or within 150 ms after target onset, the trial was aborted and not analysed. In this case, all screens briefly lit up green, followed by a 2000 ms timeout. In all cases, an inter-trial interval of 300 ms with dark screens was followed before the next trial began. Cue type, delay, target intensity and target side were balanced across conditions. Conditions were presented in a pseudo-randomized way. An automated delayed-retry protocol was used so that trials answered incorrectly would be drawn again later until all conditions were answered correctly once, and a new block was initiated. After 60 correct trials, the crows had short breaks with access to water. The task went on until the crows did not initiate any more trials or until 2 h had passed.

### (e) Task 2: central predictive cueing

The task layout is shown in figure 1*c*. Task flow was the same as in task 1, with the following differences: the precue phase always lasted 300 ms. In the cue phase, all cue stimuli were presented on the front screen. In total, 80% of all trials had a spatial cue, and 20% of all trials had a non-spatial, neutral cue. In 87.5% of spatially cued trials, a valid cue was presented 6.5 cm shifted from the centre of the screen towards the side of the upcoming target, predicting its location. In 12.5% of spatially cued trials, an invalid cue was presented 6.5 cm shifted to the side opposite to the upcoming target. Neutral cues were identical to spatial cues but appeared in the centre of the screen and did not convey spatial information. Target stimuli appeared on either side of the screen and always had a fixed intensity of 0.43 cd m^−^².

### (f) Data acquisition and analysis

We measured performance (% correct) and RT (ms) for all crows in both tasks. We recorded 25, 25, 28 and 26 sessions from Crows 1 to 4 in task 1, respectively. In addition, we recorded 25 sessions from both Crows 3 and 4 in task 2. Crows 3 and 4 had completed task 1 before being trained on task 2. Behavioural data were analysed in Matlab (MathWorks Inc., R2021b) using custom-written software.

Attentional cueing effects on RT were calculated by subtracting the median RT of all valid trials from the median RT of all invalid trials per SOA for each session. A positive difference indicates faster RTs for valid over invalid cues. To assess potential lateralization, we compared these cueing effects between left and right target trials in both tasks. In the peripheral predictive cueing task, we pooled SOAs of 200−1600 ms for each session. We excluded the shortest SOA of 100 ms to remove potential masking effects. We then split each session into three blocks containing all conditions. Because the previously reported RT effects did not depend on target intensity [13], we used intensity as a random grouping variable for each session. Median RTs for left and right targets with valid and invalid cues were calculated from these subsets and paired by intensity. This resulted in a distribution of cueing effects across sessions and intensities for both sides.

To test for differences between sides across birds, we applied a linear mixed model (LMM) to these distributions with target side as a fixed effect and bird, session and intensity as random effects (*fitlme* function in Matlab). The factors bird and intensity only have four and three levels, respectively. A low number of levels per random effect can distort the estimation of the effect’s variance, leading to recommendations for using random effects only when there is a sufficient number of levels, e.g. at least five [20]. However, recent simulation studies indicate that having fewer random effect levels does not necessarily degrade the estimation of fixed effects coefficients [21,22]. We were primarily interested in making inferences about the fixed effect of the target side while controlling for group dependencies caused by confounding background factors. Thus, following Gomes' [21] suggestion, we report the results of a full mixed model. Additionally, we controlled for the number of effect levels by comparing our results to a model with Bird and Intensity as fixed effects and Session as a random effect. This model aligned well with our initial results and did not alter our main conclusions.

For the peripheral task, a main effect of Side emerged (*t* = −3.56, d.f. = 618, *p* < 0.001), with no effects observed for either Bird (*t* = −1.89, d.f. = 618, *p* = 0.059) or Intensity (*t* = 0.42, d.f. = 618, *p* = 0.67). The Side coefficient estimates aligned well between models (${\hat{\beta }}_{1}$ = −8.16 ± 3.92 s.e., ${\hat{\beta }}_{2}$ = −8.23 ± 2.31 s.e).

For the central task, a main effect of Side was observed (*t* = −2.43, d.f. = 97, *p* = 0.017), along with an additional effect of Bird (*t* = 2.13, d.f. = 97, *p* = 0.035), indicating that Crow 3 generally showed weaker attentional modulation than Crow 4 (figure 3*c*). Again, the coefficient estimates were consistent between models (${\hat{\beta }}_{1}$ = −5.62 ± 2.31 s.e., ${\hat{\beta }}_{2}$ = −5.62 ± 2.34 s.e.).

Model parameters were fitted using maximum likelihood estimation. We further investigated the main effect of target side by comparing left and right RT effects within each crow with a paired *t*‐test.

A similar analysis was applied for the second task, the central cueing task. We pooled all SOAs and applied a LMM with target side as a fixed effect and bird and session as random effects. Cueing effects were compared within crows using a paired *t*‐test. All statistical tests had an alpha level of 5%.

## 3. Results

We conducted an experiment with four crows using two versions of a Posner-like spatial attention task. These tasks were designed with predictive cues to investigate endogenous (volitional) attention (figure 1). In this task, the crows were required to detect a faint visual target, represented by a square, as soon as it appeared on one of two side screens positioned to the left and right of the crows' centrally located head (figure 1*a*). A highly predictive cue that the crows learnt was informative and was presented before the target with a variable delay (SOA) ranging from 100 to 1600 ms, indicating the most likely location of the upcoming target. In the peripheral cueing task conducted with four crows, where the cues appeared at the same location as the impending targets, the cue accurately predicted the future target location in 89% of the trials (89% cue validity) (figure 1*b*). In the central cueing task conducted with two of the four crows, where cues appeared on a front screen and therefore at a different location than the impending target, the cue accurately predicted the target location in 87.5% of cases (figure 1*c*). The six cue-target combinations for both tasks are shown in figure 1*d*,*e*. They resulted in valid cues (cue points to the congruent target side), invalid cues (cue points to the incongruent side, opposite to where the target will appear) and neutral conditions (no cue or ambiguous cue).

**Figure 2.**
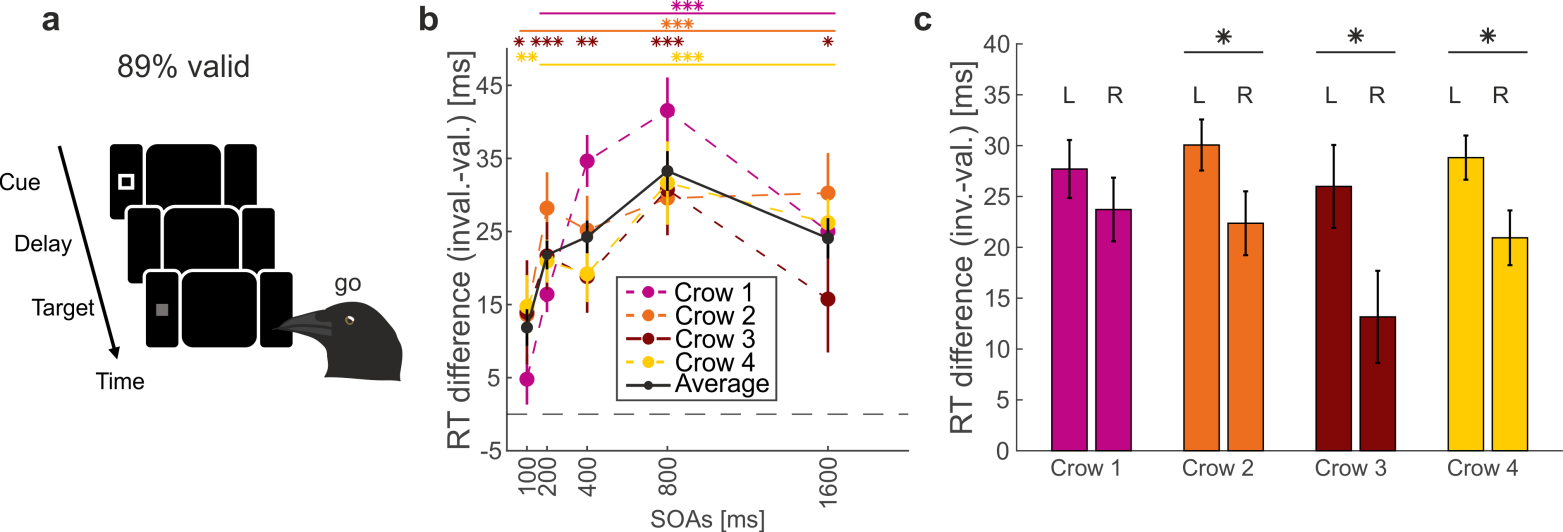
Lateralization of peripheral cueing effects. (a) Endogenous peripheral cueing task. (b) RT effects of valid versus invalid cueing across SOAs. Data from Quest *et al*. [13]. Mean RTs across sessions were significantly faster for valid than for invalid cues for all crows and SOAs (Wilcoxon signed-rank test). The black line illustrates the average RT differences for data pooled across the crows. *n* = 25, 25, 28, 26 sessions for Crows 1−4, respectively (intensities pooled). Asterisks indicate significant RT differences between valid and invalid cue trials for each individual SOA per crow. Error bars indicate the s.e.m. (c) Mean RT difference caused by endogenous attention for targets in the left (L) and right (R) monocular visual hemifield. SOAs of 200−1600 ms were pooled and each session was split into three blocks according to target intensity. RT effects were larger for left-hemifield targets in all four crows and reached significance in Crows 2, 3 and 4 (paired *t*‐test; *p* = 0.3; 0.048; 0.047; 0.019). *n* = 75, 75, 84, 78 for Crows 1−4, respectively. Error bars indicate the s.e.m. *p < 0.05, **p < 0.01, ***p < 0.001.

As reported in a previous study [13], all four crows consistently exhibited significantly faster RTs for valid cues compared to invalid cues across all SOAs in the peripheral cueing task (figure 2*a*, *n* = 25, 25, 28, 26, *p* < 0.05, Wilcoxon signed-rank test), except for Crow 1 at the shortest SOA. A positive difference indicates faster RTs for valid over invalid cues. Average RT advantages of up to 30 ms were observed (figure 2*b*). These findings demonstrate that peripheral predictive cues reliably capture endogenous attention in crows. Next, we investigated potential lateralization effects on attention by comparing RT differences, calculated as the difference between RTs during invalid trials and RTs during valid trials, for targets on both the left side (left eye) and right side (right eye).

In the peripheral cueing task, RT differences (i.e. advantages) were consistently larger by about 10 ms for left targets than for right targets across all crows (figure 2*c*). We applied a LMM to the distributions of left RT differences versus right RT differences (§2). We found a significant main effect of target side on RT differences across the crows, sessions and intensities (*n* = 624, *t* = 2.08, d.f. = 622, *p* = 0.038). RT difference was, on average, 4.0, 7.7, 12.8 and 7.9 ms larger for the left eye compared with the right eye in Crow 1−4, respectively. Thus, across all four crows, RT differences were larger for the left side compared with the right side, indicating that cue validity had a stronger effect in the left visual field. We tested this effect individually for each crow and found a significant RT difference between left and right sides for Crows 2−4 (*n* = 75; 84; 78, *p* = 0.048; 0.047; 0.019, paired *t*‐test). Crow 1 showed a RT difference effect in the same direction which did not reach significance (*n* = 75, *p* = 0.339, paired *t*‐test). In summary, RT advantages between valid and invalid cueing in the peripheral cueing task, based on endogenous attention, were more pronounced for the left visual field, indicating left lateralization.

**Figure 3.**
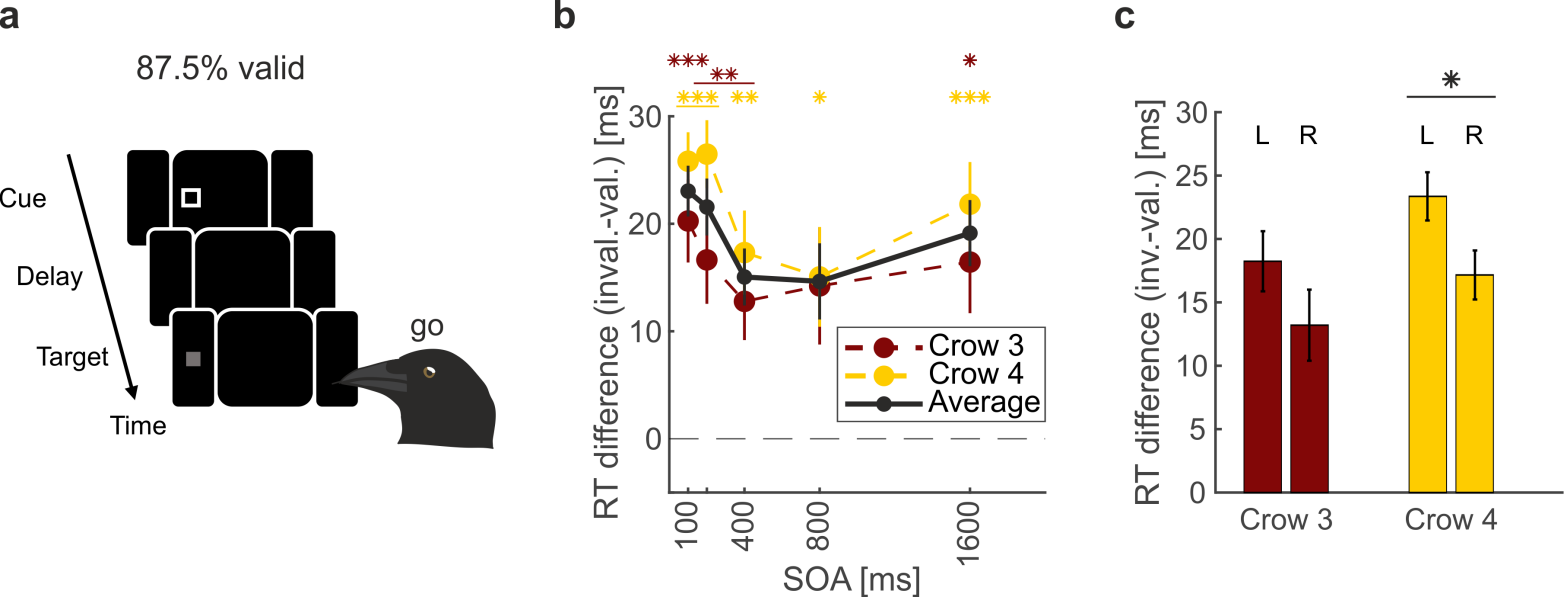
Lateralization of central cueing effects. (a) Endogenous central cueing task. (b) RT effects of valid versus invalid cueing across SOAs. Data from Hahner & Nieder [18]. Mean RTs across sessions were significantly faster for valid than for invalid cues for all crows and SOAs except SOA 800 ms in Crow 3 (Wilcoxon signed-rank test). The black line illustrates the average RT differences for data pooled across the crows. *n* = 25 sessions per crow. Asterisks indicate significant RT differences between valid and invalid cue trials for each individual SOA per crow. Error bars indicate the s.e.m. (c) Mean RT difference caused by endogenous attention for targets in the left (L) and right (R) monocular visual hemifield. All SOAs pooled. RT effects were larger for left-hemifield targets in both crows and reached significance in Crow 4 (paired *t*‐test, *p* = 0.16; 0.048). *n* = 25. Error bars indicate the s.e.m. *p < 0.05, **p < 0.01, ***p < 0.001.

In the central cueing task, cue and target locations were separated (figure 3*a*). This set-up has the advantage of excluding both potential visual masking effects and potential overlying exogenous orienting effects at short SOAs. Similar to the peripheral task, both crows tested in the central cueing task showed endogenous RT effects across SOAs (figure 3*b*). As reported in Hahner & Nieder [18], the median RTs were consistently faster for valid than for invalid cues (*n* = 25; 25, *p* < 0.05, Wilcoxon test), except for 800 ms SOA in Crow 3. On average, RTs differed by 15−25 ms across SOAs (figure 3*b*). Thus, endogenous cueing effects are preserved when the sensory appearance of the cue is separated from the target.

We additionally explored potentially lateralized attention effects in the central cueing task using the methodology described above for the peripheral cueing task. Again, RT differences were larger for left-eye than for right-eye targets (figure 3*c*). We tested for an overall side effect by applying a LMM to the distributions of left and right RT differences. There was a significant effect of target side on RT difference across crows and sessions (*n* = 100, *t* = −2.04, d.f. = 98, *p* = 0.018). RT difference was, on average, 5.0 and 6.2 ms larger for the left eye compared with the right eye in Crows 3 and 4, respectively. We tested this effect within crows and found a significant difference between sides for Crow 4 (*n* = 25, *p* = 0.048, paired *t*‐test). Crow 3 showed a non-significant difference in the same direction (*n* = 25, *p* = 0.16, paired *t*‐test). Overall, endogenous cueing effects were again left-lateralized in the central cueing task. We controlled for the low number of effect levels by comparing our results in both the peripheral cueing and central cueing tasks to a model with Bird as fixed effect and Session as random effect (§2). This control produced comparable results, confirming our main conclusions.

## 4. Discussion

Building on previous findings that crows possess robust endogenous attention [13,18], we demonstrate that their volitional attention is lateralized. In all tested crows, both the peripheral and central predictive cueing tasks revealed a systematic RT advantage for valid spatial cues when processed by the left eye (i.e. the left monocular visual field) compared with the right eye. Translated to brain processing, this suggests that the left-eye system exerts more control over volitional attention compared with the right-eye system.

We can exclude several alternative explanations for this finding. First, the observed lateralized attention effects are not influenced by basic visual factors, such as differences in how quickly crows respond to stimuli on the left versus the right or differences in the brightness of the computer displays. This is because we calculated the RT differences for valid versus invalid cues separately for each side, ensuring that any differences are due to lateralized attention and not to other variables. Second, we excluded a potential confound of overt orienting to upcoming targets by thoroughly controlling the crows’ head posture. Third, the preservation of the left-side attention advantage even with frontally placed cues in the central cueing task argues against anatomical asymmetries in the sensory organs—as observed in starlings and cockatoos [23,24]—as an explanation for the results. Therefore, our results represent a genuine side bias in top-down attention in crows.

We have direct evidence that our crows engaged in endogenous attention. In the first task, our crows demonstrated valid–invalid RT effects for peripheral predictive cues. The standard method for differentiating between endogenous and exogenous orienting typically involves contrasting central predictive cues with peripheral non-predictive cues. However, the effects of peripheral cueing can be influenced by cue predictiveness and, thus, volition. For instance, in humans, when peripheral cues are made predictive of the target, inhibition of return is abolished, indicating an endogenous modulation of the automatic orienting process [25,26]. Similar findings have been reported in monkeys using both types of peripheral cues [27].

In our previous study, we compared predictive and non-predictive peripheral cues within a controlled protocol and observed significant differences [13]. Our results showed a strong, slow and long-lasting RT effect for predictive cues, indicative of endogenous attention, compared to minimal, fast and short-lived RT effects for non-predictive cues (see fig. 5 in [13]). Furthermore, by retesting two crows in the central cue task in the current study, we were able to isolate endogenous orienting from exogenous orienting triggered by cue onset and potential visual masking of the target by the preceding stimulus at short SOAs. Although the central cues were shifted to the left or right of the frontal screen, they were always presented within the crows’ binocular field of vision, a condition ensured by our head-tracking procedure. This set-up effectively eliminated the possibility of asymmetric activation of only one eye system. In summary, the behavioural evidence confirms that our protocol effectively tested endogenous, top-down attention while controlling for other non-attentional confounds.

Lateralization phenomena can occur at both individual and population levels [28]. At the individual level, each individual of a species might show a preference for using either the left or right side. At the population level, the same side preference is consistent across many individuals within a species. In our study, the left-eye advantage in volitional attention was observed consistently across all four tested crows. While this pattern suggests a potential bias at the population level in crows, our sample size is too small to draw definitive conclusions. A different example of population-level bias is seen in New Caledonian crows. These crows demonstrate a consistent right-side preference in tool making, as evidenced by the discarded leaf tools showing a right bias across individuals [29]. In contrast, tool use in New Caledonian crows is individually lateralized, with an almost equal number showing preferences for the right or left side [30,31]. This indicates that even within the same species and behaviour, such as tool use, lateralization patterns can vary between population-level and individual-level biases.

Our finding that crows display a leftward spatial bias aligns with previously observed orientation biases in birds. Chicks and pigeons show a population-level bias for food-pecking behaviour towards the left side [4,5], and this side bias in these two bird species has been replicated ([32,33] but see Clary *et al*. [34] for indifferent side usage of black-billed magpies and Clark’s nutcrackers in the same task).

In contrast to the aforementioned overt food choice task, which does not require birds to orient based on endogenous goals as food is freely available across the test array [4,5,32], our task design specifically tests for volitional spatial attention to arbitrary stimuli based on associative cues. We therefore provide a mechanism for orientation biases in birds by showing that the executive control of attention is lateralized. This adds a top-down explanation to attentional lateralization in animals such as corvids, beyond the exogenous attention required for the food-pecking task.

In humans, the unbalanced allocation of attention across the two visual hemifields during visuospatial tasks has been known for over 40 years. In general, a similar left visual hemifield advantage (left-eye system dominance) for orienting of attention is found. Healthy humans show a phenomenon called pseudoneglect, a spontaneous tendency to attend to the left side of the visual field [35–37]. In addition, unilateral hemispatial neglect, often caused by a lesion to the right parietal cortex or underlying white matter, results in patients ignoring the left side of space [38]. More direct evidence for similar left-hemifield lateralization of endogenous spatial attention comes from a study where younger adults performed a modified Posner spatial-attentional task, similar to our task with crows [37]. This study found that younger adults responded more quickly to left-sided than right-sided cued target stimuli, demonstrating a leftward attentional bias that correlates with findings in pseudoneglect. Together, these results suggest a left-eye-system superiority for executive control of attention in humans [39].

Since both crows and humans show left-eye-system superiority for executive control, it is tempting to speculate that right-hemispheric dominance for controlling visuospatial functions might be phylogenetically conserved across species [4,40]. However, lateralization phenomena, particularly in birds, are strongly influenced by developmental effects, especially ontogenetic light experiences [41]. Visual asymmetry in birds is triggered by left-right differences in exposure of the embryo’s eyes to light in the egg, which in turn shapes visual pathways in a lateralized manner [42]. Our crows were bred and initially raised by their biological parents without manipulation, making their early ontogeny comparable to that of wild crows. Therefore, the attentional bias we observed cannot be attributed to specific rearing conditions. Moreover, the extent to which lateralized behaviour is influenced by ontogenetic light exposure in birds remains unclear. For example, a recent study found preserved lateralization of egocentric orientation in dark-incubated chicks during a spatial navigation task [43]. The precise factors underlying the lateralized executive control of attention in crows and other animals have yet to be determined.

## Data Availability

Data to reproduce all statistical analyses are published under the Open Science Framework and are available from the Dryad Digital Repository [44].

## References

[1] Güntürkün O, Ströckens F, Ocklenburg S. 2020 Brain Lateralization: A Comparative Perspective. Physiol. Rev. **100**, 1019–1063. (10.1152/physrev.00006.2019) 32233912

[2] Corballis MC. 2014 Left Brain, Right Brain: Facts and Fantasies. PLoS Biol. **12**, e1001767. (10.1371/journal.pbio.1001767) 24465175 PMC3897366

[3] Rogers LJ. 2021 Brain Lateralization and Cognitive Capacity. Animals **11**, 1996. (10.3390/ani11071996) 34359124 PMC8300231

[4] Diekamp B, Regolin L, Güntürkün O, Vallortigara G. 2005 A left-sided visuospatial bias in birds. Curr. Biol. **15**, R372–R373. (10.1016/j.cub.2005.05.017) 15916935

[5] Chiandetti C. 2011 Pseudoneglect and embryonic light stimulation in the avian brain. Behav. Neurosci. **125**, 775–782. (10.1037/a0024721) 21859172

[6] Johnen A, Wagner H, Gaese BH. 2001 Spatial Attention Modulates Sound Localization in Barn Owls. J. Neurophysiol. **85**, 1009–1012. (10.1152/jn.2001.85.2.1009) 11160532

[7] Sridharan D, Ramamurthy DL, Schwarz JS, Knudsen EI. 2014 Visuospatial selective attention in chickens. Proc. Natl. Acad. Sci. **111**, E2056–65. (10.1073/pnas.1316824111) 24753566 PMC4024881

[8] Lev-Ari T, Gutfreund Y. 2018 Interactions between top-down and bottom-up attention in barn owls (Tyto alba). Anim. Cogn. **21**, 197–205. (10.1007/s10071-017-1150-2) 29214438

[9] Lev-Ari T, Zahar Y, Agarwal A, Gutfreund Y. 2020 Behavioral and neuronal study of inhibition of return in barn owls. Sci. Rep. **10**, 7267. (10.1038/s41598-020-64197-9) 32350332 PMC7190666

[10] Posner MI. 1980 Orienting of Attention. Q. J. Exp. Psychol. **32**, 3–25. (10.1080/00335558008248231) 7367577

[11] Weichselgartner E, Sperling G. 1987 Dynamics of Automatic and Controlled Visual Attention. Science **238**, 778–780. (10.1126/science.3672124) 3672124

[12] Carrasco M. 2011 Visual attention: the past 25 years. Vis. Res. **51**, 1484–1525. (10.1016/j.visres.2011.04.012) 21549742 PMC3390154

[13] Quest M, Rinnert P, Hahner L, Nieder A. 2022 Exogenous and endogenous spatial attention in crows. Proc. Natl. Acad. Sci. **119**, e2205515119. (10.1073/pnas.2205515119) 36442123 PMC9894120

[14] Nieder A, Wagener L, Rinnert P. 2020 A neural correlate of sensory consciousness in a corvid bird. Science **369**, 1626–1629. (10.1126/science.abb1447) 32973028

[15] Nieder A. 2021 Consciousness without cortex. Curr. Opin. Neurobiol. **71**, 69–76. (10.1016/j.conb.2021.09.010) 34656051

[16] Nieder A. 2022 In search for consciousness in animals: using working memory and voluntary attention as behavioral indicators. Neurosci. Biobehav. Rev. **142**, 104865. (10.1016/j.neubiorev.2022.104865) 36096205

[17] Wagener L, Nieder A. 2024 Conscious Experience of Stimulus Presence and Absence Is Actively Encoded by Neurons in the Crow Brain. J. Cogn. Neurosci. **36**, 508–521. (10.1162/jocn_a_02101) 38165732

[18] Hahner L, Nieder A. 2023 Costs and benefits of voluntary attention in crows. R. Soc. Open Sci. **10**, 230517. (10.1098/rsos.230517) 37593715 PMC10427815

[19] Troscianko J, von Bayern AMP, Chappell J, Rutz C, Martin GR. 2012 Extreme binocular vision and a straight bill facilitate tool use in New Caledonian crows. Nat. Commun. **3**, 1110. (10.1038/ncomms2111) 23047668

[20] Arnqvist G. 2020 Mixed Models Offer No Freedom from Degrees of Freedom. Trends Ecol. Evol. **35**, 329–335. (10.1016/j.tree.2019.12.004) 31982147

[21] Gomes DGE. 2022 Should I use fixed effects or random effects when I have fewer than five levels of a grouping factor in a mixed-effects model? PeerJ **10**, e12794. (10.7717/peerj.12794) 35116198 PMC8784019

[22] Oberpriller J, de Souza Leite M, Pichler M. 2022 Fixed or random? On the reliability of mixed‐effects models for a small number of levels in grouping variables. Ecol. Evol. **12**, e9062. (10.1002/ece3.9062) 35898418 PMC9309037

[23] Hart NS, Partridge JC, Cuthill IC. 2000 Retinal asymmetry in birds. Curr. Biol. **10**, 115–117. (10.1016/s0960-9822(00)00297-9) 10662673

[24] Coimbra JP, Collin SP, Hart NS. 2014 Topographic specializations in the retinal ganglion cell layer correlate with lateralized visual behavior, ecology, and evolution in cockatoos. J. Comp. Neurol. **522**, 3363–3385. (10.1002/cne.23637) 24889497

[25] Chica AB, Bartolomeo P, Lupiáñez J. 2013 Two cognitive and neural systems for endogenous and exogenous spatial attention. Behav. Brain Res. **237**, 107–123. (10.1016/j.bbr.2012.09.027) 23000534

[26] Chica AB, Martín-Arévalo E, Botta F, Lupiáñez J. 2014 The spatial orienting paradigm: how to design and interpret spatial attention experiments. Neurosci. Biobehav. Rev. **40**, 35–51. (10.1016/j.neubiorev.2014.01.002) 24462751

[27] Fecteau JH, Bell AH, Munoz DP. 2004 Neural Correlates of the Automatic and Goal-Driven Biases in Orienting Spatial Attention. J. Neurophysiol. **92**, 1728–1737. (10.1152/jn.00184.2004) 15115792

[28] Vallortigara G. 2006 The evolutionary psychology of left and right: costs and benefits of lateralization. Dev. Psychobiol. **48**, 418–427. (10.1002/dev.20166) 16886183

[29] Hunt GR, Corballis MC, Gray RD. 2001 Laterality in tool manufacture by crows. Nature **414**, 707–707. (10.1038/414707a) 11742382

[30] Weir AAS, Kenward B, Chappell J, Kacelnik A. 2004 Lateralization of tool use in New Caledonian crows (Corvus moneduloides). Phils. Trans. R. Soc. Lond. Ser. B **271**, S344–6. (10.1098/rsbl.2004.0183) PMC1810068 15504013

[31] Martinho A III, Burns ZT, von Bayern AMP, Kacelnik A. 2014 Monocular Tool Control, Eye Dominance, and Laterality in New Caledonian Crows. Curr. Biol. **24**, 2930–2934. (10.1016/j.cub.2014.10.035) 25484292

[32] Wilzeck C, Kelly DM. 2013 Avian Visual Pseudoneglect: The Effect of Age and Sex on Visuospatial Side Biases. In Behavioral lateralization in vertebrates: two sides of the same coin (eds D Csermely, L Regolin), pp. 55–70. Berlin, Germany: Springer. (10.1007/978-3-642-30203-9_5)

[33] Chiandetti C, Galliussi J, Andrew RJ, Vallortigara G. 2013 Early-light embryonic stimulation suggests a second route, via gene activation, to cerebral lateralization in vertebrates. Sci. Rep. **3**, 2701. (10.1038/srep02701) 24048072 PMC3776965

[34] Clary D, Cheys A, Kelly DM. 2014 Pattern of visuospatial lateralization in two corvid species, black-billed magpies and Clark’s nutcrackers. Behav. Process. **107**, 94–98. (10.1016/j.beproc.2014.07.020) 25130753

[35] Jewell G, McCourt ME. 2000 Pseudoneglect: a review and meta-analysis of performance factors in line bisection tasks. Neuropsychologia **38**, 93–110. (10.1016/s0028-3932(99)00045-7) 10617294

[36] Brooks JL, Della Sala S, Darling S. 2014 Representational Pseudoneglect: A Review. Neuropsychol. Rev. **24**, 148–165. (10.1007/s11065-013-9245-2) 24414221

[37] Mańkowska A, Heilman KM, Williamson JB, Michałowski J, Harciarek M. 2020 Age-related changes in the allocation of spatially directed focal attention. Aging Neuropsychol. Cogn. **27**, 748–764. (10.1080/13825585.2019.1675581) 31610738

[38] Corbetta M, Shulman GL. 2011 Spatial Neglect and Attention Networks. Annu. Rev. Neurosci. **34**, 569–599. (10.1146/annurev-neuro-061010-113731) 21692662 PMC3790661

[39] Spagna A, Kim TH, Wu T, Fan J. 2020 Right hemisphere superiority for executive control of attention. Cortex **122**, 263–276. (10.1016/j.cortex.2018.12.012) 30661735

[40] Rogers LJ, Vallortigara G. 2015 When and Why Did Brains Break Symmetry? Symmetry **7**, 2181–2194. (10.3390/sym7042181)

[41] Güntürkün O, Ocklenburg S. 2017 Ontogenesis of Lateralization. Neuron **94**, 249–263. (10.1016/j.neuron.2017.02.045) 28426959

[42] Letzner S, Güntürkün O, Lor S, Pawlik RS, Manns M. 2017 Visuospatial attention in the lateralised brain of pigeons—a matter of ontogenetic light experiences. Sci. Rep. **7**, 15547. (10.1038/s41598-017-15796-6) 29138476 PMC5686156

[43] Morandi-Raikova A, Rosa-Salva O, Simdianova A, Vallortigara G, Mayer U. 2024 Hierarchical processing of feature, egocentric and relational information for spatial orientation in domestic chicks. J. Exp. Biol. **227**, b246447. (10.1242/jeb.246447) 38323420

[44] Hahner L, Nieder A. 2024 Data from: Volitional spatial attention is lateralized in crows. Dryad Digital Repository (10.5061/dryad.3j9kd51tm) PMC11775601 39876722

